# Comparative Genomics of Four Isosphaeraceae Planctomycetes: A Common Pool of Plasmids and Glycoside Hydrolase Genes Shared by *Paludisphaera borealis* PX4^T^, *Isosphaera pallida* IS1B^T^, *Singulisphaera acidiphila* DSM 18658^T^, and Strain SH-PL62

**DOI:** 10.3389/fmicb.2017.00412

**Published:** 2017-03-16

**Authors:** Anastasia A. Ivanova, Daniil G. Naumoff, Kirill K. Miroshnikov, Werner Liesack, Svetlana N. Dedysh

**Affiliations:** ^1^Winogradsky Institute of Microbiology, Research Center of Biotechnology of the Russian Academy of Sciences,Moscow, Russia; ^2^Max-Planck-Institute for Terrestrial Microbiology,Marburg, Germany

**Keywords:** Planctomycetes, Isosphaeraceae, *Paludisphaera borealis*, comparative genomics, plasmids, CAZy, glycoside hydrolases, glycosyltransferases

## Abstract

The family Isosphaeraceae accommodates stalk-free planctomycetes with spherical cells, which can be assembled in short chains, long filaments, or aggregates. These bacteria inhabit a wide variety of terrestrial environments, among those the recently described *Paludisphaera borealis* PX4^T^ that was isolated from acidic boreal wetlands. Here, we analyzed its finished genome in comparison to those of three other members of the Isosphaeraceae: *Isosphaera pallida* IS1B^T^, *Singulisphaera acidiphila* DSM 18658^T^, and the uncharacterized planctomycete strain SH-PL62. The complete genome of *P. borealis* PX4^T^ consists of a 7.5 Mb chromosome and two plasmids, 112 and 43 kb in size. Annotation of the genome sequence revealed 5802 potential protein-coding genes of which 2775 could be functionally assigned. The genes encoding metabolic pathways common for chemo-organotrophic bacteria, such as glycolysis, citrate cycle, pentose-phosphate pathway, and oxidative phosphorylation were identified. Several genes involved in the synthesis of peptidoglycan as well as *N*-methylated ornithine lipids were present in the genome of *P. borealis* PX4^T^. A total of 26 giant genes with a size >5 kb were detected. The genome encodes a wide repertoire of carbohydrate-active enzymes (CAZymes) including 44 glycoside hydrolases (GH) and 83 glycosyltransferases (GT) affiliated with 21 and 13 CAZy families, respectively. The most-represented families are GH5, GH13, GH57, GT2, GT4, and GT83. The experimentally determined carbohydrate utilization pattern agrees well with the genome-predicted capabilities. The CAZyme repertoire in *P. borealis* PX4^T^ is highly similar to that in the uncharacterized planctomycete SH-PL62 and *S. acidiphila* DSM 18658^T^, but different to that in the thermophile *I. pallida* IS1B^T^. The latter strain has a strongly reduced CAZyme content. In *P. borealis* PX4^T^, many of its CAZyme genes are organized in clusters. Contrary to most other members of the order Planctomycetales, all four analyzed Isosphaeraceae planctomycetes have plasmids in numbers varying from one to four. The plasmids from *P. borealis* PX4^T^ display synteny to plasmids from other family members, providing evidence for their common evolutionary origin.

## Introduction

The planctomycetes is a largely unexplored bacterial phylum that accommodates microorganisms with distinctive cell morphology and a unique cellular architecture (Schlesner and Stackebrandt, 1986; Ward, 2010; Fuerst and Sagulenko, 2011). Three distinct orders of planctomycetes are currently recognized, namely the Planctomycetales, Phycisphaerales, and Candidatus Brocadiales. The order Planctomycetales accommodates chemo-organotrophs that reproduce by budding; cells of these bacteria typically possess various multifibrillar appendages described as stalks, spikes, spines, and fimbriae (Ward, 2010). Representatives of the Phycisphaerales are also chemo-organotrophs but reproduce by binary fission (Fukunaga et al., 2009). Finally, the order Candidatus Brocadiales contains chemo-lithoautotrophic planctomycetes capable of anaerobic oxidation of ammonium (anammox; Strous et al., 1999; Jetten et al., 2009).

Planctomycetes display a number of distinctive features that are highly unusual among bacteria. Cells of planctomycetes possess a well-developed endomembrane system, which is especially pronounced in some members of the Planctomycetales (Fuerst and Sagulenko, 2011; Santarella-Mellwig et al., 2013; Devos and Ward, 2014). The ability to oxidize ammonium in anammox planctomycetes is dependent on a characteristic membrane-bound cell compartment called the anammoxosome (Neumann et al., 2014). Planctomycetes are also exceptions to the otherwise dominant mode of division by binary fission, which is based on the interaction between the FtsZ protein and the peptidoglycan (PG) biosynthesis machinery (Rivas-Marín et al., 2016). They lack a recognizable homolog of FtsZ, which plays a central role in binary fission and is conserved in almost all bacteria. PG, in its turn, has historically been thought to be absent from cell walls of planctomycetes. Recently, however, it has been demonstrated that planctomycetal genomes encode the proteins required for PG synthesis and that the cells do possess a typical PG-containing cell wall (Jeske et al., 2015; van Teeseling et al., 2015). Although the alternative division mechanism as well as the proteins involved in cell morphogenesis in planctomycetes remain enigmatic (Jogler et al., 2012; Rivas-Marín et al., 2016), these bacteria are now considered as variations of, but not exceptions to, the Gram-negative cell plan (Devos and Ward, 2014).

Planctomycetes possess large genomes, 5.5–10.1 Mb in size (Guo et al., 2014). The function, however, can be predicted for only 30–55% proteins encoded in these genomes, while the remaining proteins are usually annotated as hypothetical proteins with unknown function (Jogler et al., 2012; Guo et al., 2014). One additional feature specific for all available planctomycete genomes is the presence of so-called “giant genes” (Reva and Tümmler, 2008; Kohn et al., 2016). The majority of giant genes (with a size >5 kb) were found in non-pathogenic bacteria and annotated to encode either a cell surface protein or a non-ribosomal peptide or polyketide synthase (PK) (Reva and Tümmler, 2008). Highest numbers of giant genes (up to 60 per genome) are observed in members of the order Planctomycetales (Kohn et al., 2016).

Planctomycetes are widely distributed in various environments. As revealed by molecular surveys, these bacteria are common inhabitants of boreal *Sphagnum*-dominated peatlands (Dedysh, 2011; Ivanova and Dedysh, 2012; Serkebaeva et al., 2013; Tveit et al., 2013; Moore et al., 2015; Ivanova et al., 2016). Although several peat-inhabiting planctomycetes have been obtained in pure cultures (Dedysh and Kulichevskaya, 2013), we know very little about their potential functions in the environment. Based on our current knowledge, these bacteria are slow-acting decomposers of plant-derived organic matter. Given that most conventional tests used for assessing hydrolytic capabilities were designed for fast-growing bacteria, the analysis of degradation capabilities of peat-inhabiting planctomycetes is complicated by their slow growth rates. One of the promising strategies to circumvent these difficulties and unveil the hidden potential of slow-growing bacteria is the comparative genomic approach. For this reason, we sequenced and analyzed the genome of the recently described planctomycete *Paludisphaera borealis* PX4^T^, which was isolated from boreal *Sphagnum* peat bog of northern Russia (Kulichevskaya et al., 2016). This bacterium is a chemo-organotrophic, mildly acidophilic, and psychrotolerant aerobe. Cells of this planctomycete are Gram-negative, non-motile spheres that occur singly or in short chains.

The apparent ability of hydrolyzing gellan gum (a complex heteropolysaccharide of microbial origin; Phytagel^TM^), as well as several other polysaccharides, makes *P. borealis* PX4^T^ an attractive target for genome-based studies on its carbohydrate metabolism. Carbohydrate-active enzymes (CAZymes) are responsible for synthesis and degradation of oligo- and polysaccharides as well as their derivatives. CAZymes include glycoside hydrolases (GH), glycosyltransferases (GT), polysaccharide lyases (PL), and carbohydrate esterases (CE). Based on homology of the catalytic domains, they form about 250 protein families, including 129 families of glycoside hydrolases and transglycosidases (GH1–GH135, except for GH21, GH40, GH41, GH60, GH61, and GH69) (Lombard et al., 2014; Terrapon et al., 2017). The CAZyme repertoire of microorganisms is strongly determined by the ecological niche they occupy (Naumoff, 2011b).

**FIGURE 1:**
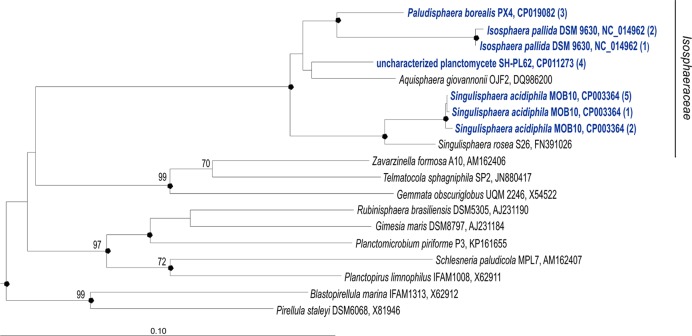
**16S rRNA gene-based neighbor-joining tree (Jukes–Cantor correction) showing the phylogenetic position of *Paludisphaera borealis* PX4^T^ in relation to the three Isosphaeraceae reference organisms used in this study, i.e., *Isosphaera pallida* IS1B^T^, *Singulisphaera acidiphila* DSM 18658^T^, and undescribed planctomycete strain SH-PL62S (shown in blue).** All 16S rRNA gene copies revealed in the genomes of these four planctomycetes are included in the tree. The number of identical 16S rRNA gene copies is shown in parenthesis. Different 16S rRNA gene copies of *S. acidiphila* DSM 18658^T^ are grouped as follows (locus-tags are given): *S. acidiphila* (5)—Sinac_3310, Sinac_3707, Sinac_3773, Sinac_5935, Sinac_6238; *S. acidiphila* (2)—Sinac_2684, Sinac_4695; *S. acidiphila* (1)—Sinac_5862. Different 16S rRNA gene copies of *I. pallida* IS1B^T^ are grouped as follows (locus-tags are given): *I. pallida* (2)— Isop_R0040, Isop_R0051; *I. pallida* (1)—Isop_R0061. The significance levels of interior branch points obtained in neighbor-joining analysis were determined by bootstrap analysis (1000 data re-samplings). Bootstrap values (1000 data resamplings) of ≥70% are shown. Black circles indicate that the corresponding nodes were also recovered in the maximum-likelihood and maximum-parsimony trees. The root (not shown) was composed of five 16S rRNA gene sequences from anammox planctomycetes (AF375994, AF375995, AY254883, AY257181, and AY254882). Bar, 0.1 substitutions per nucleotide position.

*Paludisphaera borealis* PX4^T^ is a member of the family Isosphaeraceae in the order Planctomycetales (Kulichevskaya et al., 2016). This family accommodates stalk-free planctomycetes with spherical cells, which can be assembled in short chains, long filaments, or shapeless aggregates. Other described genera in this family are *Isosphaera*, *Singulisphaera*, and *Aquisphaera*. At present, complete genome sequences are available for three members of this family, i.e., *Isosphaera pallida* IS1B^T^, *S. acidiphila* DSM 18658^T^, and the uncharacterized planctomycete strain SH-PL62 (**Figure 1**). Comparative genomic analysis between *P. borealis* PX4^T^ and the other three members of the Isosphaeraceae revealed remarkable similarity in their genome organization. All four planctomycetes harbor plasmids which share multiple homologous regions. Many genes in *P. borealis* PX4^T^ have their closest homologs in strain SH-PL62 and *S. acidiphila* DSM 18658^T^. Here, we present the genomic characteristics of *P. borealis* PX4^T^ together with a detailed description of its CAZyme repertoire.

## Materials and Methods

### Cultivation Procedures

*Paludisphaera borealis* PX4^T^ (= DSM 28747^T^ = VKM B-2904^T^) was grown in shaking liquid cultures at 24°C in M2 medium of the following composition (g l^-1^ distilled water): KH_2_PO_4_, 0.1; (NH_4_)_2_SO_4_, 0.1; MgSO_4_ × 2H_2_O, 0.1; CaCl_2_ × 2H_2_O, 0.02; yeast extract (Difco), 0.02; glucose (Sigma), 0.5; mineral salt solution “44,” 1 ml (Staley et al., 1992); pH 5.5–5.8. After 3 weeks of incubation, the biomass was collected and transferred to the Max-Planck Genome Centre Cologne (MP-GCC, Germany) for DNA extraction, library preparation, and sequencing.

To verify the presence of several hydrolytic capabilities predicted by genome analysis, we performed additional substrate utilization tests as outlined in the original description of *P. borealis* (Kulichevskaya et al., 2016). For these tests, glucose was omitted from medium M2 and the latter was supplemented with 0.05 % (w/v) of the corresponding substrate. The growth was examined by measuring the rate of CO_2_ production in tightly closed 120 ml flasks containing 10 ml liquid medium M2 with tested substrate for 3 weeks at 24°C. Control incubations were run in parallel under the same conditions but without substrate.

### Genome Sequencing

Genome sequencing of strain PX4^T^ was performed at the MP-GCC, using the PacBio RSII platform with a single SMRT^®^ cell (Pacific Biosciences, Menlo Park, CA, USA). A total of 56,515 sequences were obtained with a mean length of 9279.29 bp (9.28 kb) [total length = 524,419,229 bp or 524,419 kb; N50 value = 12,777 bp (12.78 kb)]. *De novo* assembly was done using the hierarchical genome-assembly process (HGAP2) via the SMRT Portal v.2.0 offered by Pacific Biosciences (Chin et al., 2013). The parameter settings were as follows: (i) minimum subread length: 500; (ii) minimum polymerase read quality: 0.8; (iii) minimum polymerase read length: 100; (iv) minimum seed read length: 6000; (v) overlapper error rate: 0.06; (vi) minimum overlapping length: 40; and (vii) overlapping K-mer: 14. The draft assembly was manually checked and redundant terminal sequences were removed.

### Genome Annotation

Initial automated genome annotation was carried out using *RAST* v. 2.0 (Rapid Annotation using Subsystem Technology) with default parameters (Aziz et al., 2008; Overbeek et al., 2014; Brettin et al., 2015). Subsequent inspection was done in *PROKKA* package (Seemann, 2014) including all dependencies such as *PRODIGAL* v 2.6.2 (Hyatt et al., 2010), *HMMER* server (Finn et al., 2011), *RNAMMER* (Lagesen et al., 2007), *BLAST+* (Camacho et al., 2009), and *ARAGORN* (Laslett and Canback, 2004). Annotation with *PROKKA* was performed against both the *UNIPROT* database (Apweiler et al., 2009) and a manually constructed database that includes all available annotated planctomycete genome sequences. Their visualization was done in *BRIG* program (Alikhan et al., 2011).

The analysis of *P. borealis* PX4^T^ genome sequence using the Kyoto Encyclopedia of Genes and Genomes (KEGG) was performed applying GhostKOALA tool (Kanehisa et al., 2016). Screening for secondary metabolite-related genes was performed using the online web server antiSMASH3.0.5 (Antibiotics & Secondary Metabolites Analysis Shell; Medema et al., 2011; Blin et al., 2013; Weber et al., 2015).

The automated annotation of CAZymes by *RAST* v. 2.0 was manually checked in order to validate their affiliation to CAZy (Lombard et al., 2014) and PFAM (Finn et al., 2016) families. Additionally, all Isosphaeraceae proteins listed in the CAZy database were used as queries for blastp searches of the *P. borealis* PX4^T^ proteome. Representatives of each GHL (Naumoff, 2011a, 2016; Naumoff and Stepuschenko, 2011), FURAN (Naumoff, 2012), PFAM (Naumoff, 2011b), and COG (Naumoff, 2011b) family of putative glycoside hydrolases were used as queries, as well. Identified CAZymes were analyzed for both possible alternative start-codons and domain structure. Predicted catalytic domains were used as queries for blastp searches in an iterative manner. The dbCAN server (Yin et al., 2012) was used to classify all obtained proteins into existing CAZy families. Remaining unclassified proteins were analyzed and annotated manually. Each gene encoding an incomplete catalytic domain was considered a pseudogene.

The annotated genome sequence of *P. borealis* PX4^T^ has been deposited in the GenBank database under BioProject number PRJNA354242 (sequence accession numbers CP019082-CP019084).

### Phylogenetic Analyses

Phylogenetic analysis of 16S rRNA gene sequences from *P. borealis* PX4^T^ and other representative members of the order Planctomycetales was carried out using the *ARB* program (Ludwig et al., 2004). The significance levels of interior branch points obtained in the neighbor-joining analysis were determined by bootstrap analysis (based on 1000 data re-samplings). The stability of various nodes were also confirmed with *PHYLIP* maximum-likelihood and maximum-parsimony methods (Felsenstein, 1989) implemented in *ARB* package.

### Comparative Genomics

In addition to *P. borealis* PX4^T^, the finished genome sequences of three other Isosphaeraceae planctomycetes were used for comparative analysis (NCBI accession number in parenthesis): *I. pallida* IS1B^T^ (BioProject No PRJNA32825), *S. acidiphila* DSM 18658^T^ (PRJNA52461), and the uncharacterized planctomycete strain SH-PL62 (PRJNA277747) (**Figure 1**). The overall similarities between the genome of *P. borealis* PX4^T^ and the three reference genomes were estimated using average nucleotide identity (ANI) calculator and formula 2 of the Genome-to-Genome-Distance-Calculator (Auch et al., 2010a,b) under parameter settings proposed elsewhere (Meier-Kolthoff et al., 2013).

## Results

### Genome Properties

**FIGURE 2:**
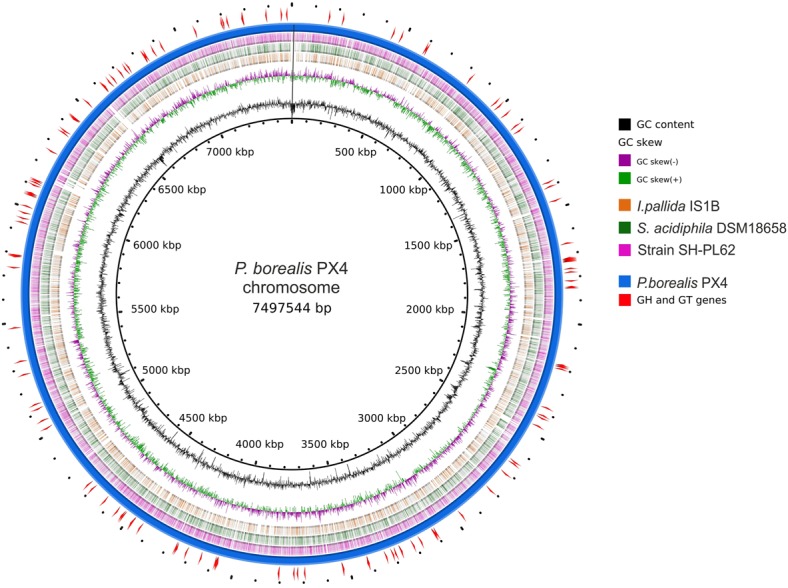
**Scheme of *Paludisphaera borealis* PX4^T^ genome.** The innermost circle gives the genome coordinates. The next two inner rings display the GC skew and the G + C content along the genome. Next three rings indicate the chromosome regions with nucleotide sequence identity of 70–100% (based on blastn) between *P. borealis* PX4^T^ and the three Isosphaeraceae reference organisms: *Isosphaera pallida* IS1B^T^, *Singulisphaera acidiphila* DSM 18658^T^, and strain SH-PL62, respectively. The outermost two rings show the chromosome of *P. borealis* PX4^T^ and the positions of genes encoding glycoside hydrolases and glycosyltransferases.

**FIGURE 3:**
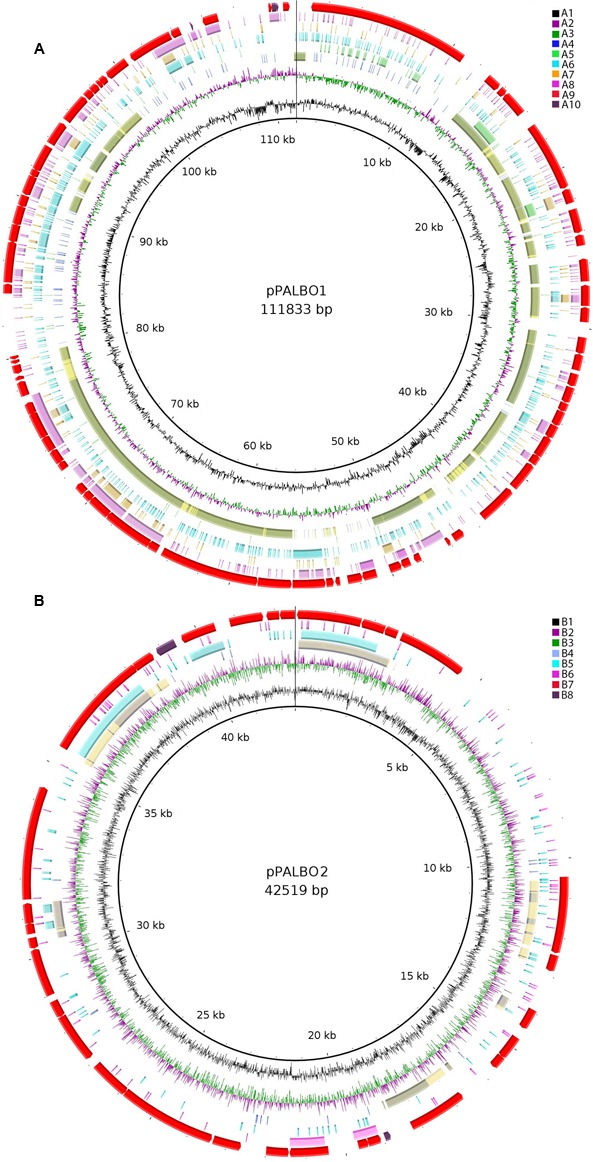
**(A)** Comparative genomic analysis of plasmid pPALBO1. The blastRing Image Generator (BRIG) software was used to compare pPALBO1 with nearest homologs. Different genome sequences, indicated by colors within concentric circles, are as follows: A1, GC content; A2 and A3, GC skew (+, green; −, purple); A4, plasmid pPL62-1 of strain SH-PL62 (GenBank accession CP011274.1) (blue—sequence similarity according to BRIG; yellow—manual prediction; green—both); A5, plasmid pSINAC01 (GenBank accession CP003365.1) from *Singulisphaera acidiphila* DSM 18658^T^; A6, chromosome of strain SH-PL62 (GenBank accession CP011273.1); A7, chromosome of *Isosphaera pallida* IS1B^T^ (GenBank accession CP002353.1); A8, sequence similarity to the chromosome of *S. acidiphila* DSM 18658^T^ (GenBank accession CP003364.1); A9, location of genes on plasmid pPALBO1; A10, pseudogenes. **(B)** Comparative genomic analysis of plasmid pPALBO2. The BRIG software was used to compare pPALBO2 with nearest homologs. Different genome sequences, indicated by colors within concentric circles, are as follows: B1, GC content; B2 and B3, GC skew (+, green; −, purple); B4, plasmid pSINAC03 from *S. acidiphila* DSM 18658^T^ (blue—sequence similarity according to BRIG; yellow—manual prediction; green—both); B5, chromosome of strain SH-PL62; B6, chromosome of *S. acidiphila* DSM 18658^T^; B7, location of genes on plasmid pPALBO2; B8, pseudogenes.

The genome of *P. borealis* PX4^T^ consists of a 7.498 Mb chromosome and two plasmids, a large plasmid of 111.833 kb and a small plasmid of 42.519 kb (**Tables 1, 2** and **Figures 2, 3**). The GC content of the chromosomal DNA is 66.3%. The corresponding values for the large and small plasmids are 65.3% and 59.3%, respectively. All three sequences (chromosome and plasmids) are finished and have no gaps. Three copies of 16S-23S-5S rRNA operon and 79 tRNA genes were identified. Annotation of the genome sequence revealed 5802 potential protein-coding genes of which 2775 could be functionally assigned. The genome contains a single Clustered Regularly Interspaced Short Palindromic Repeats (CRISPR) locus and a set of CRISPR-associated (cas) genes.

**Table 1:** Genome statistics for planctomycetes of the family Isosphaeraceae.

Features	*P. borealis* PX4^T^	*S. acidiphila* DSM 18658^T^	Strain SH-PL62	*I. pallida* IS1B^T^
Genome size (Mb)	7.652	9.742	6.785	5.529
Chromosome (Mb)	7.498	9.630	6.507	5.473
Number of plasmids	2	3	4	1
GC content (%)	66.26	63.01	65.56	62.4
Total genes	5 890	7 516	5 271	4 005
CDS	5 802	7 363	4 941	3 770
rRNA operons	3	8	4	3
tRNA genes	79	64	53	47
CRISPR repeats	1	5	1	2

**Table 2:** Plasmid overview.

Organism	Plasmids	Size (bp)	GC (%)	Genes
*Paludisphaera borealis* PX4^T^	PALBO1	111,833	65.3	73
	PALBO2	42,519	59.3	32
*Singulisphaera acidiphila* DSM 18658^T^	SINAC01	54,731	60.1	29
	SINAC02	39,149	59.9	51
	SINAC03	32,131	59.0	31
Planctomycete SH-PL62	PL62-1	97,874	69.02	77
	PL62-2	86,054	67.74	65
	PL62-3	81,115	69.41	58
	PL62-4	12,514	66.67	18
*Isosphaera pallida* IS1B^T^	ISOP01	56,340	67	32

The genome characteristics of *P. borealis* PX4^T^ in comparison to those of *I. pallida* IS1B^T^, *S. acidiphila* DSM 18658^T^, and strain SH-PL62 are summarized in **Table 1**. The genome size in these bacteria varies from 5.529 Mb in thermophilic *I. pallida* IS1B^T^ to 9.742 Mb in *S. acidiphila* DSM 18658^T^. The G + C content range within the Isosphaeraceae is 62.4–66.3 mol%; it is lowest in *I. pallida* IS1B^T^ and highest in *P. borealis* PX4^T^. The number of ribosomal operons is highest in *S. acidiphila* DSM 18658^T^ (eight), which also possesses greatest number of CRISPR repeats (five).

### Phylogenetic Analyses

No sequence differences were observed between the three 16S rRNA gene copies present in the genome of *P. borealis* PX4^T^ (**Figure 1**). The same was true for the four 16S rRNA gene copies in the genome of strain SH-PL62. Two of the three 16S rRNA gene copies in *I. pallida* IS1B^T^ are also identical but display two mismatches to the third copy. Finally, eight copies of 16S rRNA gene sequences present in the genome of *S. acidiphila* DSM 18658^T^ can be divided into three types, containing five, two, and one sequence(s), respectively. These three sequence types, however, differ by one to four nucleotide positions only. The use of one or another sequence type for phylogenetic inference does not affect the tree topology.

The phylogenetic position of *P. borealis* PX4^T^ relative to the other Isosphaeraceae planctomycetes is shown in **Figure 1**. *P. borealis* PX4^T^ displayed 91 and 92% 16S rRNA gene sequence similarity to *I. pallida* IS1B^T^ and *S. acidiphila* DSM 18658^T^, respectively. The corresponding similarity value to the 16S rRNA gene sequence of strain SH-PL62 is 95%. This strain forms a separate phylogenetic lineage and, most likely, represents a novel genus within the Isosphaeraceae, despite its relatively high 16S rRNA gene similarity with *P. borealis* PX4^T^.

*Paludisphaera borealis* PX4^T^ shares the following overall genome similarities with the three reference organisms: 20.2 ± 2.2% (*S. acidiphila* DSM 18658^T^); 19.8 ± 2.3% (*I. pallida* IS1B^T^); and 16.7 ± 2.3% (strain SH-PL62). These DNA–DNA hybridization values were estimated using formula 2 of the Genome-to-Genome-Distance-Calculator. They are in the range generally calculated for members of different genera (Scheuner et al., 2014). Accordingly, the ANI values shared between the genomes of *P. borealis* PX4^T^ and the other three Isosphaeraceae are also very low: 77% (*S. acidiphila* DSM 18658^T^); 75% (*I. pallida* IS1B^T^); and 73% (strain SH-PL62).

### Metabolism and Cell Biology

KEGG-based annotation of *P. borealis* PX4^T^ genome sequence classified 1784 proteins into 17 major functional categories. The annotated genomes of *S. acidiphila* DSM 18658^T^, *I. pallida* IS1B^T^, and strain SH-PL62 were available via the KEGG database. The distribution of genes among the major KEGG categories was similar in all four examined planctomycete genomes, with slight variations in gene numbers in some of the categories (**Supplementary Figure S1**).

The genes encoding metabolic pathways common for chemo-organotrophic bacteria, such as glycolysis, the citrate cycle, the pentose-phosphate pathway, and oxidative phosphorylation were present in the genome of *P. borealis* PX4^T^. This planctomycete has the genomic potential for synthesis of all amino acids. The number of ABC-transporters in *P. borealis* PX4^T^ (35) is somewhat smaller than those identified in other members of the Isosphaeraceae (40 in *I. pallida* IS1B^T^ to 48 in *S. acidiphila* DSM 18658^T^). These numbers are comparable to the calculated mean of 49 ABC-transporters in free-living prokaryotes (Glöckner et al., 2003). Also, two fructose-type sugar-specific subunits of phospho-transferase system could be found in strain PX4^T^.

Most genes essential for chemotaxis were identified in the genome of *P. borealis* PX4^T^. These include *cheA*, *cheB*, *cheR*, and *cheW*. Similar gene arrays were present in *S. acidiphila* DSM 18658^T^ and strain SH-PL62, but only *cheW* was identified in *I. pallida* IS1B^T^. Poor representation of genes responsible for flagellar assembly in the four examined planctomycetes agrees well with the fact that all described members of the family Isosphaeraceae do not produce motile swarmer cells as typical for planctomycetes from other families of the order Planctomycetales.

The survey for genes related to cell division revealed a situation characteristic of other planctomycetes (Rivas-Marín et al., 2016). Namely, the FtsZ-encoding gene was absent, while two copies of the gene coding for FtsK, the DNA translocase, were present in the genome of *P. borealis* PX4^T^. The cytoskeletal protein MreB whose gene has a patchy presence among the planctomycetes and is absent in *S. acidiphila* DSM 18658^T^ (Rivas-Marín et al., 2016), is also missing in the other three members of the Isosphaeraceae. Several but not all genes involved in PG biosynthesis, including *murB*, *murE*, and *mraY*, were detected.

A few years ago, the presence of novel *N*-methylated ornithine membrane lipids (OLs) was reported for several peat-inhabiting planctomycetes, including *S. acidiphila* DSM 18658^T^ (Moore et al., 2013). OLs are phosphorus-free membrane lipids widespread in bacteria but absent from archaea and eukaryotes. Recently, the gene encoding the key enzyme for synthesis of *N*-methylated OLs, *N*-methyltransferase (OlsG), was identified in the genome of *S. acidiphila* DSM 18658^T^ (Sinac_1600; Escobedo-Hinojosa et al., 2015). We revealed homologs of Sinac_1600 in the genomes of *P. borealis* PX4^T^ and the other two Isosphaeraceae members studied here as well as in the genomes of two Gemmataceae planctomycetes, *Zavarzinella formosa* A10^T^ and *Gemmata* sp. SH-PL17. This suggests that *N*-methylation of OLs is a common trait among members of these planctomycete families.

Finally, *P. borealis* PX4^T^ and *S. acidiphila* DSM 18658^T^ possessed the greatest genetic potential for biosynthesis of secondary metabolites and antibiotics among the four Isosphaeraceae planctomycetes (**Supplementary Figure S1**).

### CAZyme Repertoire

Homology analysis of all proteins potentially encoded in the genome of *P. borealis* PX4^T^ was performed in order to reveal the complete set of CAZymes. As a result, 44 glycoside hydrolases, 83 glycosyltransferases, and 12 carbohydrate esterases belonging to, respectively, 21, 13, and 8 CAZy families were detected (**Table 3** and Supplementary Tables S1–S3). The CAZyme repertoire in *P. borealis* PX4^T^ was highly similar to those in strain SH-PL62 and *S. acidiphila* DSM 18658^T^, but different to that in *I. pallida* IS1B^T^ (**Table 3**). The latter organism is thermophilic and has a strongly reduced CAZyme content, but the enzymes belong mainly to the same protein families as those from the other Isosphaeraceae planctomycetes. Additionally, we predict a significant number of proteins, which do not belong to any of the currently recognized CAZy families (Lombard et al., 2014) but display a distant relationship to some glycoside hydrolases, glycosyltransferases, or carbohydrate esterases (Supplementary Tables S4–S6). The majority of CAZymes from *P. borealis* PX4^T^ was most closely related to those identified in strain SH-PL62 (Supplementary Tables S1–S6).

**Table 3:** Families of carbohydrate-active enzymes represented in *P. borealis* PX4^T^ (1), *S. acidiphila* DSM18658^T^ (2), *I. pallida* IS1B^T^ (3), and planctomycete SH-PL62 (4).

CAZy family	1	2	3	4	CAZy family	1	2	3	4
GH1	0	1	0	0	GH129	0	0	0	1
GH2	1	2	0	1	GH130	2^∗^	4	0	2
GH5	4	2	1	5	GT1	1	3	0	1
GH9	1	0	1	1	GT2	32	40	24	32
GH10	1	1	1	1	GT4	38^∗^	53	35	32
GH13	9^∗^	10	7	9	GT5	1	0	1	1
GH15	1	2	2	2	GT9	3	3	3	3
GH17	0	0	1	0	GT19	1	1	1	1
GH18	1	1	0	1	GT21	1	1	1	1
GH25	0	1	0	0	GT25	1	0	0	1
GH29	1	0	1	2	GT26	1	0	0	1
GH32	2	1	0	2	GT27	0	1	0	0
GH33	3	3	1	8	GT28	0	1	0	0
GH38	1	0	0	0	GT30	1	1	1	1
GH39	0	0	0	2	GT35	1	1	1	1
GH43	3	2	0	3	GT41	0	2	1	0
GH50	0	0	0	1	GT71	0	0	1	0
GH51	1	0	0	1	GT75	0	0	0	1
GH57	4	3	2	4	GT76	0	1	0	0
GH73	0	1	0	0	GT83	4	6	2	5
GH77	1	1	0	1	GT84	1	1	0	1
GH78	2	0	0	2	CE1	1	4	1	1
GH88	1	1	1	0	CE4	1	0	0	0
GH94	2	1	0	2	CE6	1	1	0	1
GH95	1	1	0	1	CE9	1	1	1	1
GH97	0	1	0	0	CE11	1	1	1	1
GH104	0	1	0	0	CE14	5	5	0	4
GH116	0	0	0	1	CE15	1	1	1	1
GH127	2	0	0	2	CE16	1	0	0	1

*Data for (2–4) are given according to the CAZy database (Lombard et al., 2014).*

*^∗^Numbers indicated by asterisks include some pseudogenes (see Supplementary Tables S1, S2).*

Many CAZyme genes of *P. borealis* PX4^T^ are organized in clusters. We identified nine gene clusters, each containing at least three glycosyltransferase and/or glycoside hydrolase genes (not shown). Two largest clusters include eight (BSF38_01353–BSF38_01364) and five (BSF38_01716–BSF38_01727) glycosyltransferase genes. The third cluster is composed of three genes coding for GH13-family proteins (BSF38_02409, BSF38_02411, BSF38_02412). One of the remaining clusters is located on plasmid pPALBO1 (see below). One of two isoamylase genes (BSF38_01066 and BSF38_01066a) is partly duplicated in the form of tandem repeat (Supplementary Table S1). In addition, some other CAZyme genes are also present in the form of pseudogenes (see notes to **Table 3**).

### Genome-Predicted versus Expressed Hydrolytic Capabilities

According to the original description (Kulichevskaya et al., 2016), *P. borealis* is capable of hydrolyzing aesculin, cellobiose, gellan gum, lactose, lichenin, maltose, melibiose, pectin, salicin, sucrose, trehalose, and xylan, while chondroitin sulfate and raffinose are not utilized. A large proportion of the experimentally determined substrate utilization pattern could be confirmed by the genome-derived data. A phenotypic trait that draw our attention to this planctomycete, namely the formation of visible depressions in gellan-solidified media (Kulichevskaya et al., 2016), is in agreement with the presence of genes encoding two α-L-rhamnosidases (GH78) and an unsaturated glucuronyl hydrolase (GH88). Utilization of aesculin, cellobiose, lactose, lichenin, maltose, salicin, trehalose, and xylan can be explained by the occurrence of genes encoding GH2, GH5, GH9, GH13, GH15, GH43, and GH51-domain containing proteins. The use of sucrose as growth substrate is in line with the encoded β-fructosidase (BSF38_03534). A substrate, which was not tested in the original study but suggested by the genome analysis to be utilized, is arabinan. The inability to utilize chondroitin sulfate can be explained by the absence of genes known to be responsible for its degradation: hyaluronidase (GH16, GH56, GH84), chondroitin hydrolase (GH56), hyaluronate lyase (PL8, PL16), chondroitin AC lyase (PL8), or chondroitin ABC lyase (PL8).

The earlier reported abilities of growth on melibiose and pectin, however, could not be confirmed by the genome data. The genes coding for polygalacturonase (GH28), pectate lyase (PL1, PL2, PL3, PL10), exo-pectate lyase (PL1, PL2), pectin lyase (PL1), pectin methylesterase (CE8), or pectin acetylesterase (CE12, CE13) were lacking, thereby suggesting inability of pectin utilization by *P. borealis* PX4^T^. We also did not identify genes encoding CAZymes of the GH4, GH27, GH31, GH36, GH97, or GH110 families. These are the only families known to contain bacterial α-galactosidases (Lombard et al., 2014). Inability to utilize raffinose and the absence of a secreted α-galactosidase activity (according to API ZYM test; see Kulichevskaya et al., 2016) also supported the dubiety of the earlier reported melibiose utilization by *P. borealis*. One additional uncertainty between the genome-predicted and experimentally determined capabilities of strain PX4^T^ was the presence of a putative chitinase from GH18 family and the lack of experimental evidence for its growth on chitin.

These analyses prompted us to verify several genome-predicted capabilities in *P. borealis* PX4^T^, i.e., the ability to grow on arabinan and chitin. In addition, we reassessed its ability to develop on melibiose, pectin, and raffinose. With the exception that growth on chitin could not be demonstrated, the results of these experiments were in full agreement with the genome-derived data.

### Plasmids

All four Isosphaeraceae planctomycetes harbor plasmids. Their numbers vary from one in *I. pallida* IS1B^T^ to four in strain SH-PL62 (**Table 2**). *P. borealis* PX4^T^ possesses two plasmids, pPALBO1 (large plasmid) and pPALBO2 (small plasmid).

pPALBO2 display synteny to plasmid pSINAC03 from *S. acidiphila* DSM 18658^T^. Their common evolutionary origin is obvious despite the fact that pPALBO2 is a bit larger (**Table 2**). These two plasmids have 10 regions of homology and possess 12 orthologous genes located exactly in the same order and orientation (**Figure 3B**). Each of the proteins encoded by these genes in *P. borealis* PX4^T^ finds its counterpart in *S. acidiphila* DSM 18658^T^ as the best hit in blastp search. One of them is ParB-like nuclease (BSF38_20001). The closest homologs of three other pPALBO2 genes (BSF38_20020, BSF38_20021, BSF38_20023) are located on the chromosome of *S. acidiphila* DSM 18658^T^ (**Figure 3B**). However, BSF38_20023 homolog (Sinac_1451) is annotated as a pseudogene. The gene BSF38_20023 also has a more distant homolog located on plasmid pSINAC03 (Sinac_7675). One additional pPALBO2 gene (BSF38_20034) has a distant homolog in pSINAC03 (Sinac_7679) which is also annotated as a pseudogene. The latter two genes of pPALBO2 (BSF38_20023 and BSF38_20034) differ in their position and orientation from their counterparts in pSINAC03, suggesting an independent evolutionary history. Notably, nine genes located on pPALBO2 have close homologs on the chromosome of strain SH-PL62. All of them are located within the same 23 kb locus. Thus, it is tempting to conclude that a pPALBO2-like plasmid has been integrated into a chromosome of strain SH-PL62 ancestor.

The large plasmid pPALBO1 from *P. borealis* PX4^T^ displays high similarity to plasmid pPL62-1 from strain SH-PL62. The two plasmids have 28 regions of homology and 51 orthologous genes in common (**Figure 3A**). Most likely, two of these genes are pseudogenes in the case of pPL62-1 (data not shown). The order of genes is essentially the same in both plasmids except for a long inversion involving 14 common genes. One of the homologous loci includes the cluster of glycosyltransferase genes mentioned above. It encodes proteins from the GT4 and GT26 families as well as two proteins from the GT2 family (Supplementary Table S2). Two other homologous loci contain a gene for phosphorylase from the GH94 family (Supplementary Table S1) and ParA ATPase (BSF38_10004). Two genes present in both plasmids, pPALBO1 and pPL62-1, also have homologs on plasmid pSINAC01 from *S. acidiphila* DSM 18658^T^. These two genes encode a replication initiator protein A (BSF38_10006) and a glucose-1-phosphate thymidylyltransferase (BSF38_10008). Plasmids pPALBO1 and pSINAC01 have an additional pair of homologous genes (BSF38_10002) coding for a putative β-propeller-type glycoside hydrolase (Supplementary Table S4), which does not belong to any of the currently recognized CAZy families (Lombard et al., 2014). Plasmids pPL62-1 and pSINAC01 also have an additional pair of homologous genes (VT85_25820) encoding a DUF1559-containing protein. Twenty-three of 51 genes shared by pPALBO1 and pPL62-1 have homologs on the chromosome of *S. acidiphila* DSM 18658^T^, but these are widely distributed over the chromosome. Thus, there may already be a long history of independent evolution between the gene homologs in pPALBO1/pPL62-1 and those in *S. acidiphila* DSM 18658^T^.

### Giant Genes

The examination of *P. borealis* PX4^T^ genome for the presence of giant genes (Reva and Tümmler, 2008) revealed 26 genes with a size >5 kb, among which only one gene exceeds 10 kb. Two giant genes (BSF38_10002 and BSF38_10039; Supplementary Tables S1, S4) are plasmid-borne (pPALBO1) and appear to be responsible for carbohydrate metabolism, while all the others are located on the chromosome. The genomes of *S. acidiphila* DSM 18658^T^ and *I. pallida* IS1B^T^ contain 36 and 16 genes, respectively, with a size >5 kb (Kohn et al., 2016). Strain SH-PL62 possesses 23 giant genes. For comparison, the highest number (60) of giant genes with a size >5 kb was detected in the genome of *Z. formosa* A10^T^, while the largest giant genes (with a size around 36 kb) were identified in “*Fuerstia marisgermanica*” NH11^T^ and *Gimesia maris* DSM 8797^T^ (Kohn et al., 2016).

### Secondary Metabolites-Related Genes

**FIGURE 4:**
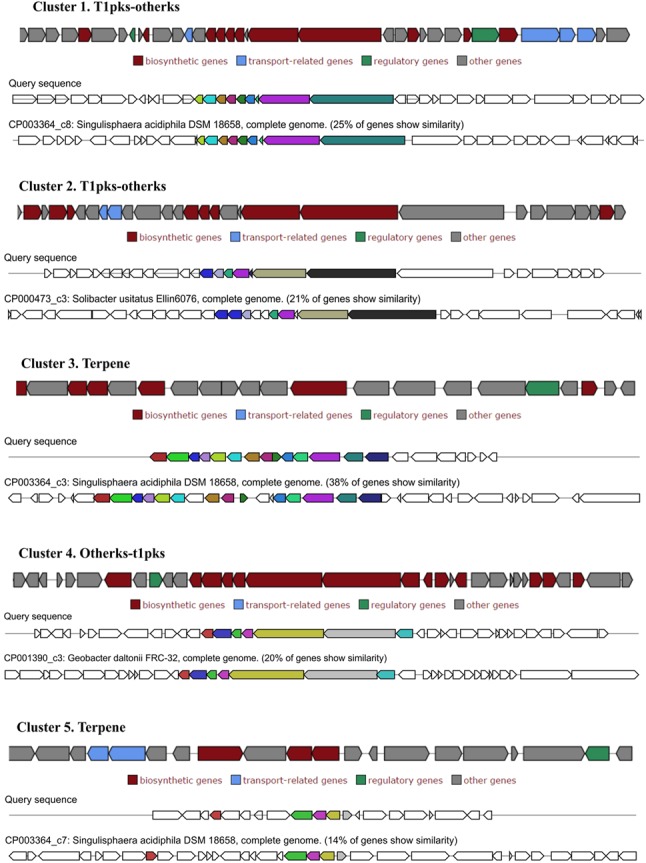
**Secondary metabolite-related gene clusters identified in the genome of *Paludisphaera borealis* PX4^T^ with antiSMASH and displayed with homologous gene clusters from other bacteria**.

Genome mining of gene clusters that encode biosynthetic pathways for secondary metabolites in *P. borealis* PX4^T^ revealed five clusters that comprise 137 genes (**Figure 4**). The gene clusters 1, 2, and 4 encode PKS of type I and others (t1pks and others, respectively). Closest gene homologs to PKS-encoding gene clusters from *P. borealis* PX4^T^ belong to *S. acidiphila* DSM 18658^T^, “*Solibacter usitatus*” Ellin6076, and *Geobacter daltonii* FRC-32 (**Figure 4**). The gene clusters 3 and 5 encode terpenes. Their closest homologs belong to *S. acidiphila* DSM 18658^T^.

## Discussion

Our analysis revealed remarkable similarity in genome organization between members of the family Isosphaeraceae. All four analyzed representatives of this family have plasmids in numbers varying from one to four and sizes varying from 13 to 112 kb. Notably, the presence of plasmids has not yet been reported for any other described member of the order Planctomycetales, with one exception: a 37-kb plasmid in *Planctopirus limnophila* (Labutti et al., 2010). It should be noted, however, that only finished genomes without gap allow the conclusive identification of plasmids. The lack of reports on the presence of plasmids in Planctomycetales other than Isosphaeraceae has therefore to be interpreted with care. As shown in **Figure 3**, a number of plasmid regions in the four studied Isosphaeraceae members display synteny, providing evidence for their common evolutionary origin. The small plasmid pPALBO2 from *P. borealis* PX4^T^ and the plasmid pSINAC03 from *S. acidiphila* DSM 18658^T^ display 10 regions of homology and share 12 orthologous genes. Interestingly, some of these genes are also present on the chromosome of strain SH-PL62, where they are organized in a tight cluster that, most likely, originated via plasmid integration into the chromosome. The large plasmid from *P. borealis* PX4^T^, pPALBO1, carries an array of glycosyltransferase genes (families GH94, GT2, GT4, and GT26) and, in addition, two genes (BSF38_10002 and BSF38_10010) encoding CAZymes that display a distant relationship to those currently listed in the CAZy database (Lombard et al., 2014). Plasmid pPALBO1 shows high similarity to pPL62-1 from strain SH-PL62. Some of the genes common to both plasmids, pPALBO1 and pPL62-1, have homologs on plasmid pSINAC01 from *S. acidiphila* DSM 18658^T^. Apparently, the large plasmids in mesophilic Isosphaeraceae planctomycetes encode parts of the enzyme machinery required for carbohydrate biosynthesis.

One additional observation of interest is that the genes encoding ATPase ParA and nuclease ParB, both involved in DNA partitioning, are located on different plasmids in strain PX4^T^ (pPALBO1 and pPALBO2, respectively). The activity of both proteins is required for proper distribution of each plasmid replicate to the daughter cells of *P. borealis* PX4^T^ during cell division. Given this fact, the expression of ATPase ParA and nuclease ParB on the two different plasmids may thus be the reason why both plasmids, pPALBO1 and pPALBO2, are stably maintained in *P. borealis* PX4^T^, despite the increased metabolic cost.

It is well known that the CAZyme repertoire in a particular organism reflects its lifestyle and ecology and also depends on its genome size (Coutinho and Henrissat, 1999; Henrissat et al., 2002; Coutinho et al., 2003; Naumoff, 2011b). The major glycoside hydrolase families in *P. borealis* PX4^T^ are GH5 (contains retaining enzymes with various β-glycopyranosidase activities), GH13 (retaining α-glycopyranosidases), and GH57 (retaining α-glucopyranosidases). Genome analysis allowed us to conclude that cells of *P. borealis* PX4^T^ may possess α-L-arabinofuranosidase, β-L-arabinofuranosidase, chitinase, cyclic β-1,2-glucan synthetase, β-fructofuranosidase, α-L-fucosidase, 1,4-α-glucan branching, β-glucanase, 4-α-glucanotransferase, α-glucosidase, isoamylase, malto-oligosyltrehalose synthase, malto-oligosyltrehalose trehalohydrolase, α-mannosidase, phosphorylase, α-L-rhamnosidase, sialidase, trehalose synthase, and unsaturated glucuronyl hydrolase activities (Supplementary Table S1). In general, the genome-predicted spectrum of substrates utilized by *P. borealis* PX4^T^ was in agreement with that reported in the original taxonomic description (Kulichevskaya et al., 2016). Several corrections in the list of growth substrates, however, have to be made. As suggested by the genome analysis and confirmed by cultivation experiments, arabinan should be included in the list of potential growth substrates, while melibiose and pectin should be excluded. These results clearly demonstrate the strength of genome-based predictions.

In the genome of *P. borealis* PX4^T^, we also identified several dozens of genes that encode proteins which cannot be affiliated with the currently recognized CAZy families (Lombard et al., 2014). They, however, display a distant relationship to some glycoside hydrolases, glycosyltransferases, or carbohydrate esterases (Supplementary Tables S4–S6). Particularly, we detected eighteen β-propeller-fold proteins that are homologous to an unclassified β-galactosidase from an uncultured bacterium (GenPept, AGW45552.1). This group of proteins is distantly related to the FURAN31 family of putative glycoside hydrolases (Naumoff, 2012). We also identified 20 DUF1080-containing proteins; two of these consist of two homologous domains. According to the PFAM database, the DUF1080 family is related to the GH16 family of glycoside hydrolases and belongs to the same clan (Finn et al., 2016). In addition, we detected forty one PF01408-containing proteins. In the CAZy database, many proteins of this family are classified as “glycoside hydrolases not yet assigned to a family” (also known as the GH_NC family). Given this unexpectedly large number of unclassified putative glycoside hydrolases, we conclude that *P. borealis* PX4^T^ has an extremely high but partly hidden glycolytic potential.

**FIGURE 5:**
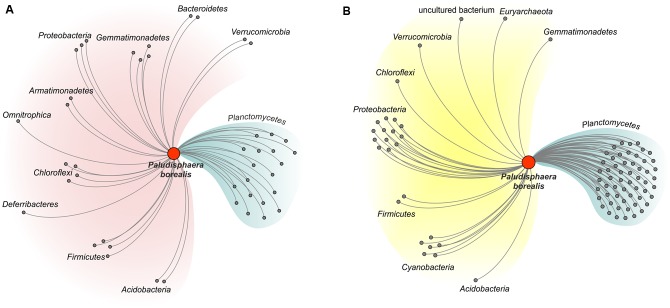
**Network diagram illustrating the proposed evolutionary origin of the genes encoding glycoside hydrolases (A)** and glycosyltransferases **(B)** in *Paludisphaera borealis* PX4^T^. The diagram was built for proteins of *Paludisphaera borealis* PX4^T^ based on the list of closest homologs as shown in the column “Others” in Supplementary Tables S1, S2 (the orthologs from *S. acidiphila* DSM 18658^T^ and strain SH-PL62 were ignored). CAZymes of *Paludisphaera borealis* PX4^T^ with closest homologs in prokaryotic phyla other than Planctomycetes were considered as transferred horizontally. The proteins are grouped according to the host phylum.

The three mesophilic Isosphaeraceae planctomycetes examined in our study, i.e., *P. borealis* PX4^T^, *S. acidiphila* DSM 18658^T^, and strain SH-PL62, appear to possess a common CAZyme pool. Indeed, 86% of the glycoside hydrolases identified in *P. borealis* PX4^T^ have their closest homologs in strain SH-PL62; one-third of these enzymes was also detected in *S. acidiphila* DSM 18658^T^ (Supplementary Table S1). Similarly, 76 and 65% of the glycosyltransferases identified in *P. borealis* PX4^T^ have very close homologs in strain SH-PL62 and *S. acidiphila* DSM 18658^T^, respectively (Supplementary Table S2). We made the attempt to trace the evolutionary origin of the enzymes present in *P. borealis* PX4^T^, including those shared with *S. acidiphila* DSM 18658^T^ and strain SH-PL62, by examining the taxonomic source of their closest homologs (**Figure 5**; see organisms listed in the column “Others” in Supplementary Tables S1, S2). Apparently, a major proportion of the genes coding for glycoside hydrolases (57%) and glycosyltransferases (33%) were acquired by lateral gene transfer from other phyla, such as Proteobacteria, Chloroflexi, Cyanobacteria, Gemmatimonadetes, Verrucomicrobia, and Acidobacteria. Surprisingly, CAZyme genes from *P. borealis* PX4^T^ had only a very few closest homologs with members of the Firmicutes and Bacteroidetes, the two bacterial phyla known for their high hydrolytic potential. Even more surprising, the CAZymes from *P. borealis* PX4^T^ did not share any closest homolog with the Actinobacteria, the third group of hydrolytic bacteria whose genomes are overrepresented in the GenBank database. Apparently, the CAZyme repertoire in *P. borealis* PX4^T^ and closely related planctomycetes significantly differs from those in well-studied hydrolytic bacteria.

We also analyzed the genome-encoded potential of *P. borealis* PX4^T^ for secondary metabolite production. As it was shown previously, the ability to produce secondary metabolites is a “luxury” that only large-genome bacteria can afford (Donadio et al., 2007). Moreover, the bacterial genomes in general and planctomycete genomes in particular display a linear correlation between genome size and their capacity to encode putative secondary metabolites (Donadio et al., 2007; Jeske et al., 2013). Among the finished Isosphaeraceae genomes, *S. acidiphila* DSM 18658^T^ has the largest one and the highest number of secondary metabolite-related gene clusters (11) (see Jeske et al., 2013). By contrast, the genome of *I. pallida* IS1B^T^ is rather small (**Table 1**) and encodes only five secondary metabolite gene clusters (Jeske et al., 2013). The genome of strain SH-PL62 contains two terpene-encoding gene clusters, three gene clusters of PKS type I, and one gene cluster of PKS type 3. In our study, we revealed three PKS gene clusters and two terpene-encoding gene clusters in *P. borealis* PX4^T^. Terpenes are a diverse class of organic compounds that include different kinds of antibiotics, hormones, flavor components, vitamins, and pigments (Yamada et al., 2012). The planctomycete genomes are known to be extremely rich in terpenoid gene clusters (Jeske et al., 2013). Since many planctomycetes, including *P. borealis* PX4^T^, form pink-colored colonies, carotenoid synthesis might account for several planctomycetal terpenoid synthases (Jeske et al., 2013). PKS are multimodular enzymes that direct the formation of oligopeptide and polyketide secondary metabolites on a protein template. Their specific multimodular molecular assembly allows the PKS to produce numerous small bioactive compounds that can be used as antimicrobials, plant protectives, or nematicides (Fischbach and Walsh, 2006; Walsh, 2008; Jeske et al., 2013). Given that several planctomycete PKS gene clusters or genes are rather small, one of their functional roles could be in modifying the ribosomally synthesized bacteriocins and lantibiotics (Cotter et al., 2013; Jeske et al., 2013).

In summary, comparative genomics revealed high glycolytic potential in *P. borealis* PX4^T^, which remains to be explored in future studies. Many enzymes in the CAZyme pool of strain PX4^T^ are shared with other mesophilic Isosphaeraceae planctomycetes, providing these bacteria with the ability to utilize a wide range of natural carbohydrates and glycoconjugates. The potential to produce a range of secondary metabolites is another characteristic of *P. borealis* PX4^T^ that deserves additional attention.

## Author Contributions

SD, AI, and WL designed the study. AI cultivated the strain. AI, DN, and KM analyzed and interpreted the sequence data. AI and DN annotated the genome sequence. DN analyzed the repertoire of carbohydrate-active enzymes. DN and KM performed the comparative analysis of plasmids. SD, AI, DN, and WL wrote the manuscript.

## Conflict of Interest Statement

The authors declare that the research was conducted in the absence of any commercial or financial relationships that could be construed as a potential conflict of interest.

## Supplementary Material

FIGURE S1 **The heatmap summarizes the functional annotation of protein-coding genes from *Paludisphaera borealis* PX4^T^, *Singulisphaera acidiphila* DSM 18658^T^, *Isosphaera pallida* IS1B^T^, and strain SH-PL62 using KEGG database.** Only the functional categories with >10 assignments for at least one organism is shown.
