# Supplementary material for: Comparative Genomics of Four Isosphaeraceae Planctomycetes: A Common Pool of Plasmids and Glycoside Hydrolase Genes Shared by Paludisphaera borealis PX4T, Isosphaera pallida IS1BT, Singulisphaera acidiphila DSM 18658T, and Strain SH-PL62
Source: Front Microbiol. 2017 Mar 16;8:412. doi: 10.3389/fmicb.2017.00412 (PMC5352709; doi:10.3389/fmicb.2017.00412)
Supplement: FIGURE S1 — The heatmap summarizes the functional annotation of protein-coding genes from Paludisphaera borealis PX4T, Singulisphaera acidiphila DSM 18658T, Isosphaera pallida IS1BT, and strain SH-PL62 using KEGG database. Only the functional categories with >10 assignments for at least one organism is shown. [file Image_1.pdf]

	P. borealis	*PL-SH62*	*I. pallida*	*S.acidiphila*
**Global and overview maps**				
01100 Metabolic pathways	460	419	399	483
01110 Biosynthesis of secondary metabolites	214	203	190	214
01120 Microbial metabolism in diverse environments	144	119	103	137
01130 Biosynthesis of antibiotics	160	146	132	159
01200 Carbon metabolism	90	73	68	78
01210 2-Oxocarboxylic acid metabolism	21	20	20	20
01212 Fatty acid metabolism	17	14	10	17
01230 Biosynthesis of amino acids	89	85	80	91
01220 Degradation of aromatic compounds	6	5	3	4
**Carbohydrate metabolism**				
00010 Glycolysis / Gluconeogenesis	26	23	20	25
00020 Citrate cycle (TCA cycle)	22	17	17	22
00030 Pentose phosphate pathway	20	19	14	21
00040 Pentose and glucuronate interconversions	12	12	10	13
00051 Fructose and mannose metabolism	17	17	12	18
00052 Galactose metabolism	11	8	7	13
00500 Starch and sucrose metabolism	17	16	11	18
00520 Amino sugar and nucleotide sugar metabolism	28	28	25	32
00620 Pyruvate metabolism	28	22	21	28
00630 Glyoxylate and dicarboxylate metabolism	27	21	21	19
00640 Propanoate metabolism	22	16	17	17
00650 Butanoate metabolism	13	11	10	13
**Energy metabolism**				
00190 Oxidative phosphorylation	38	39	36	41
00710 Carbon fixation in photosynthetic organisms	12	12	9	13
00720 Carbon fixation pathways in prokaryotes	29	20	21	24
00680 Methane metabolism	25	22	19	20
00910 Nitrogen metabolism	15	16	7	15
00920 Sulfur metabolism	14	15	11	19
**Lipid metabolism**				
00061 Fatty acid biosynthesis	12	11	9	13
00564 Glycerophospholipid metabolism	15	18	9	15
**Nucleotide metabolism**				
00230 Purine metabolism	51	40	39	55
00240 Pyrimidine metabolism	35	30	31	36
**Amino acid metabolism**				
00250 Alanine, aspartate and glutamate metabolism	21	21	22	22
00260 Glycine, serine and threonine metabolism	27	25	21	24
00270 Cysteine and methionine metabolism	24	25	23	29
00280 Valine, leucine and isoleucine degradation	14	8	10	9
00290 Valine, leucine and isoleucine biosynthesis	10	10	10	10
00300 Lysine biosynthesis	9	10	7	9
00220 Arginine biosynthesis	16	11	10	15
00330 Arginine and proline metabolism	14	12	10	14
00400 Phenylalanine, tyrosine and tryptophan biosynth	18	19	17	18
00450 Selenocompound metabolism	11	6	9	12
**Glycan biosynthesis and metabolism**				
00540 Lipopolysaccharide biosynthesis	19	10	13	19

00550 Peptidoglycan biosynthesis	4	15	3	9
**Metabolism of cofactors and vitamins**				
00760 Nicotinate and nicotinamide metabolism	12	11	12	13
00770 Pantothenate and CoA biosynthesis	15	13	14	15
00790 Folate biosynthesis	15	13	13	16
00670 One carbon pool by folate	12	11	9	12
00860 Porphyrin and chlorophyll metabolism	18	17	21	32
00130 Ubiquinone and other terpenoid-quinone biosyn	10	10	11	12
**Metabolism of terpenoids and polyketides**				
00900 Terpenoid backbone biosynthesis	11	10	10	12
**Translation**				
03010 Ribosome	51	51	47	51
00970 Aminoacyl-tRNA biosynthesis	24	25	25	25
**Folding, sorting and degradation**				
03060 Protein export	11	12	10	11
03018 RNA degradation	13	14	12	12
**Replication and repair**				
03030 DNA replication	12	12	8	14
03410 Base excision repair	11	10	7	13
03430 Mismatch repair	15	13	11	17
03440 Homologous recombination	16	16	12	21
**Membrane transport**				
02010 ABC transporters	35	42	40	48
03070 Bacterial secretion system	16	13	9	17
**Signal transduction**				
02020 Two-component system	42	38	28	50
**Cell motility**				
02030 Bacterial chemotaxis	10	11	3	12
02040 Flagellar assembly	8	11	8	10
**Cellular community**				
02024 Quorum sensing	17	26	15	18
